# Evaluation of Binding of Rosmarinic Acid with Human Transferrin and Its Impact on the Protein Structure: Targeting Polyphenolic Acid-Induced Protection of Neurodegenerative Disorders

**DOI:** 10.1155/2020/1245875

**Published:** 2020-11-05

**Authors:** Anas Shamsi, Saleha Anwar, Mohd Shahbaaz, Taj Mohammad, Mohamed F. Alajmi, Afzal Hussain, Imtaiyaz Hassan, Faizan Ahmad, Asimul Islam

**Affiliations:** ^1^Centre for Interdisciplinary Research in Basic Sciences, New Delhi, India; ^2^South African National Bioinformatics Institute, University of the Western Cape, Private Bag X17, Bellville, Cape Town 7535, South Africa; ^3^Laboratory of Computational Modeling of Drugs, South Ural State University, 76 Lenin Prospekt, Chelyabinsk, 454080, Russia; ^4^Department of Pharmacognosy, College of Pharmacy, King Saud University, Riyadh 11451, Saudi Arabia

## Abstract

Rosmarinic acid (RA) is a natural compound that is gaining wide popularity owing to its broad-spectrum biological activities. RA is known for its wide range of medicinal properties and therapeutic applications in a vast range of neurodegenerative disorders thus making it a vital natural compound. Human transferrin (hTf) is a clinically significant protein that plays a pivotal role in maintaining iron homeostasis. The importance of studies pertaining to hTf is attributable to the pivotal role of iron deposition in CNS in neurodegenerative disorders. The study was intended to have an insight into the interaction between RA and hTf employing multispectroscopic approach, molecular docking, and molecular dynamic simulation studies. Fluorescence quenching studies revealed that RA shows an excellent binding affinity to hTf with a binding constant (*K*) of 10^7^ M^−1^ and is guided by static mode of quenching. Isothermal titration calorimetry (ITC) further validated the spontaneous nature of binding. The estimation of enthalpy change (∆*H*) and entropy change (∆*S*) suggested that the RA-hTf complex formation is driven by hydrogen bonding, thereby making this process seemingly specific. Further, Fourier transform infrared (FTIR) and circular dichroism (CD) spectra suggested that RA induces conformational and structural changes in hTf. Additionally, molecular dynamics (MD) studies were carried out to investigate the stability of the hTf and hTf–RA system and suggested that binding of RA induces structural alteration in hTf with free hTf being more stable. This study provides a rationale to use RA in drug development against neurodegenerative disorders by designing novel functional foods containing RA.

## 1. Introduction

The importance of natural compounds dates to ancient times as many of these have potential therapeutic applications. In this growing disease era, where each day an individual is diagnosed with a new disease, the significance of these natural compounds has increased [1]. Studies about natural compounds are on a high in recent times [2] due to vital properties possessed by them. Polyphenols are one of the most investigated classes of natural compounds extensively present in plants and foods like herbs, nuts, vegetables, fruits, and plant-derived beverages like tea and coffee [3–6]. The importance of polyphenols can be attributed to their broad-spectrum therapeutic potentials in different diseases and hence are being employed for drug discovery [6–9].

Amongst naturally occurring polyphenolic compounds, phenolic acids are secondary plant metabolites that are taken up in the diet frequently [10]. Rosmarinic acid (RA) (Figure SIA) is a phenolic compound generally found in various plants from the Lamiaceae (the mint) family. The importance of studies related to RA depends on the fact that it is associated with several diverse activities such as antioxidative, anticancer, and many more [11]. RA is increasingly being used in the cosmetic industry [12] and the food industry [13] and thus is an important compound to be studied. Rosmarinic acid is used for food preservation, i.e., to garnish and improve the shelf life of seafood [14]. RA is now known to be one of the most promising food-functional polyphenols, thus highlighting its significance.

In the human body, several essential elements are playing a vital role in having diverse functions. Iron is one such element, and its homeostasis is maintained by transferrin and ferritin as iron in free form is a potent neurotoxin [15]. Transferrin family is a group of proteins having a task of transporting iron around the bloodstream postforming an iron-protein complex [16]. Human transferrin (hTf) is a glycoprotein having 679 amino acid residues and has a molecular mass of 79.6 kDa [17] (Figure SIB). Native hTF is folded in a manner that creates a cleft that provides an encouraging environment for the binding of Fe3+. Fe3+ is bound octahedrally to the side chains of Tyr95, Tyr188, Asp63, His249, and two oxygen from carbonate [18]. The importance of this protein can be owed to the fact that many studies report excessive iron deposition in the central nervous system (CNS) in neurodegenerative pathologies, namely, Alzheimer's disease (AD) and Parkinson's disease (PD) [19].

Many studies report the formation of complexes as a result of interaction between ligand and proteins [15], and this complex formation leads to modification of structure and properties of both ligand and protein that are involved in the interaction. Post binding of the ligand to protein, conformational alterations in proteins can occur which further affect their functionality [20]. Thus, it is imperative to study the binding of the ligand with protein in a bid to elucidate their mechanism of action in the body. It is imperative to have an insight into the behavior and action of RA specifically to be aware of its transport and distribution properties in the circulatory system. Its behavior in the circulatory system can be affected by its binding with plasma proteins. As a result of interaction with plasma proteins, with significant advancements in the pharmaceutical industry, the detailed insight of interaction between plasma proteins and drugs is a vital step in pharmacological profiling thus making this study clinically significant.

Our present study was targeted to delineate the interaction between RA and hTf *in vitro* and *in silico* and to understand the interaction mechanism for hTf-RA interaction. It was expected that RA will bind strongly with plasma protein, hTf, and induce structural changes in hTf, or, in contrast, RA will bind to hTf with weak affinity and does not induce any structural alterations in native conformation of hTf. Fluorescence spectroscopy, isothermal titration calorimetry (ITC), and quenching studies were carried out to have an insight into the binding nature and mechanism of hTf-RA interaction. Further, RA-induced conformational changes in hTf were investigated by circular dichroism (CD) and Fourier transform infrared (FTIR) spectroscopy. Additionally, *in vitro* observations were further validated by molecular docking and molecular dynamic simulation (MD) studies. The study can serve as an important asset for the food industry in the treatment of neurodegenerative disorders by targeting the designing of novel functional foods containing RA and its derivatives.

## 2. Materials and Methods

### 2.1. Materials

Human transferrin and RA were purchased from Sigma-Aldrich Co. (St. Louis, MO, USA). All other chemicals required for buffer preparation were obtained from HiMedia. The stock solution of hTf (5 mg/ml) was prepared in 20 mM sodium phosphate buffer, and the pH 7.4. RA stock solution (1 mg/ml) was made in distilled water. All the solutions were kept in the dark at 4°C before use. Appropriate blanks were used as control and run under the same conditions, and the reported spectra here are subtracted spectra. A through degassing was carried out for all the samples.

### 2.2. Fluorescence Spectroscopy

Jasco FP 6200 (Japan) was used to measure the fluorescence spectra of native hTf and hTf in the presence of RA (4-36 *μ*M) and analyzed as described earlier [21, 22] making use of the Stern-Volmer equation, modified Stern-Volmer equation, and van't Hoff equation [23].

### 2.3. Circular Dichroism (CD) Spectroscopy and Fourier Transform Infrared (FTIR) Spectroscopy

CD spectra of free protein and protein in the presence of RA were recorded making use of the JASCO-J1500 spectropolarimeter connected with a Peltier-temperature controller in the range of 200-250 nm at room temperature under a nitrogen atmosphere with a slit width of 2 nm. Each spectrum has an average of five scans. The experimental parameters were as follows: recording range was 200-250 nm, the scan rate of 100 nm/min with a response time of 1 s, and 1 mm quartz cell was used [17]. The concentration of protein was 6 *μ*M while the concentration of RA was 54 *μ*M. FTIR spectra measurements were done using Agilent Technologies Carry 630 FTIR fitted to a MICROTEK with an MCT detector. The protein concentration was 8 *μ*M while 72 *μ*M RA was taken, in the ratio of 1 : 9.

### 2.4. Isothermal Titration Calorimetry (ITC)

ITC measurements were done at 25°C on a VP-ITC microcalorimeter from MicroCal, Inc (GE, MicroCal, USA) as per previously published studies [21]. There is a programmed titration with the first false injection of 2 *μ*l and subsequent 10 *μ*l injections at 260 seconds interval carrying ligand were titrated into the main protein present in the sample cell. hTf was present in the sample cell (20 *μ*M) with RA (200 *μ*M) in the syringe. The stirring rate of the injector was kept at 320 rpm. The heat of dilution of the ligand in buffer was subtracted from the titration data. MicroCal Origin 8.0 was used to calculate the stoichiometry of binding (*n*), enthalpy change (Δ*H*), and association constant (*K*_a_).

### 2.5. UV–Vis Absorption Spectroscopy

The UV–Vis absorption measurements were carried out using a Jasco F-660 UV spectrometer in a 1.0 cm quartz cuvette at room temperature. All samples were incubated for 30 min, and the spectra were recorded in the range of 240-340 nm.

### 2.6. Molecular Docking Analysis

DELL ® Workstation with 4x 2.13 GHz processor, 64 GB RAM, and two TB hard disks running on the Ubuntu 14.04.5 LTS operating system was retorted for molecular docking analysis. For carrying out docking coupled with visualization, online resources such as Protein Data Bank (PDB) and PubChem were used in retrieval of the three-dimensional coordinates of hTf and RA. Bioinformatic tools AutoDock Vina [24], Discovery studio [25], and PyMOL [26] were employed for docking and visualization purposes.

Atomic coordinates of hTf were taken from the PDB (ID: 3 V83) and subsequently preprocessed in SPDBV [27] and AutoDock Tools [28]. Subsequently, cocrystal ligand and water were removed from the coordinate file. RA was obtained from the PubChem database in a three-dimensional format and processed in AutoDock Tools. The docking was structurally blind for the compound where it was free to be in motion and search the binding site(s) of the protein. The search space for RA was set to a grid size of 58, 88, and 72 Å, centralized at -51.31, -1.13, and -30.17 for *X*, *Y*, and *Z* coordinates on hTf, respectively. The grid spacing was set to 1.00 Å with the exhaustiveness of 8. In total, nine docked conformations were obtained, out of which the one based on interaction and binding affinity was selected.

### 2.7. Molecular Dynamic Simulations (MD)

MD simulations on the RA bound and unbound form of hTf were performed using GROMACS version 2018-2 [29]. Primarily, the GROMOS96 53a6 force field [30] was used for the generation of topologies of the protein structure in the docking-based generated complexes. Moreover, the topologies of the studied ligand compound were generated using the PRODRG server [31], but the PRODRG server does not contain the functionality of generating the partial charges of the RA; therefore, the DFT method implemented in Gaussian which utilized the B3LYP 6-31G (d,p) basis set and the CHELPG program [32] was used for the charge correction. After successful topology generation of the docked complexes, they were solvated using the SPC/E water model [33] and then neutralized by adding a suitable number of NA and CL counter ions. Consequently, the system was subjected to energy minimization step using combined steepest descent as well as conjugate gradient algorithms, with a convergence criterion of 0.005 kcal/mol. Before the equilibration step, the position restraints were applied to the structure of the ligand in the minimized system.

The equilibration step was carried out into the combined stages of NVT (constant volume) and NPT (constant pressure) ensemble conditions, each at 100 ps time scale. The temperature of 300 K was maintained for the system using the Berendsen weak coupling method, and the pressure of 1 bar was maintained utilizing Parrinello-Rahman barostat in the equilibration stage. The LINCS algorithm was used for the generation of the final conformational production stage for 100 ns time scale, and trajectories were generated, which were analyzed to understand the behavior of each complex in the explicit water environment. The changes in the protein-ligand distance, H-bonds, RMSD, *R*_g,_ RMSF, PCA, and free energy landscapes of the complex system were analyzed. Furthermore, the molecular mechanics Poisson–Boltzmann surface area (MM-PBSA) protocols implemented in the g_mmpbsa package [34] was used for the calculation of free energy of binding protein and the ligand molecules.

## 3. Results and Discussion

### 3.1. Binding Analysis of RA with hTf

#### 3.1.1. Fluorescence-Based Binding Studies

Quenching of fluorescence is an event where fluorescence of a protein is quenched in the presence of a quencher. Fluorescence quenching can either be static or dynamic or a combination of static and dynamic [35]. Static quenching is that event in which there is a ground-state complex formation between fluorophore and quencher while dynamic quenching is that event where the collision between fluorophore and quencher occurs in an excited state.

The operative mode of quenching for specific drug-protein interaction can be found out making use of temperature dependency of the quenching process [36], i.e., the variation of Stern-Volmer constant and the biomolecular quenching rate constant with temperature. Thus, fluorescence quenching experiments were carried out at three different temperatures and quenching data was analyzed by employing different mathematical equations, namely, Stern-Volmer, double log relation, and van't Hoff equation.

Stern-Volmer equation (equation (1)) and the modified Stern-Volmer equation (equation (2)) were employed to analyze the fluorescence quenching data at three different temperatures to find different binding parameters.

(1) $$\begin{array}{c} \frac{F_{0}}{F}=1+K_{\text{sv}}\left[C\right], \end{array}$$

(2) $$\begin{array}{c} \log\frac{F_{0}-F}{F}=\log K+n\log\left[C\right]. \end{array}$$

**Figure 1:**
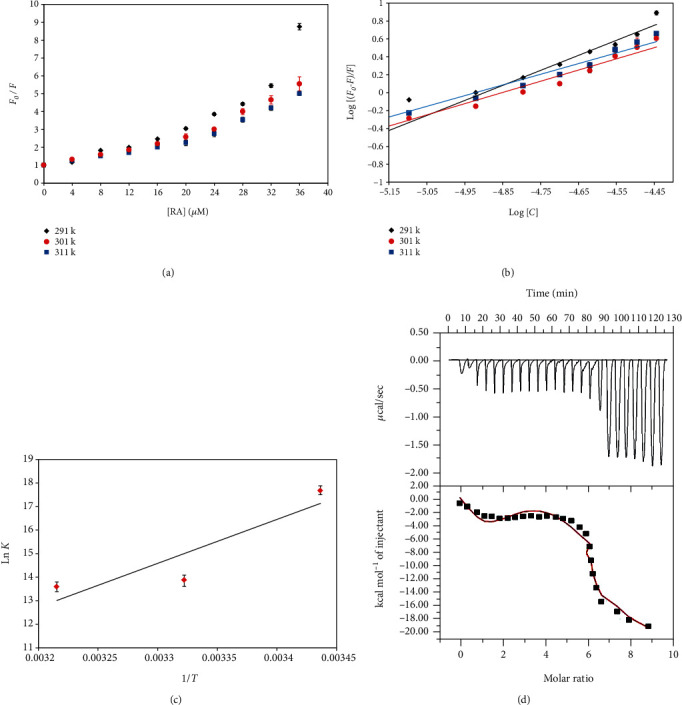
(a) Stern–Volmer plots for the quenching of hTf fluorescence by RA at three different temperatures. (b) Modified Stern-Volmer plot. The experimental data fitting of double log relation at three different temperatures. (c) van't Hoff plot with the natural log of obtained binding constant at three different temperatures with the inverse of retorted temperatures on the *x*-axis. (d) ITC isotherm obtained after titrating RA with hTf. The four-site fit curve is displayed as a thin line. Experiments were done in triplicate.

Figure 1(a) shows the Stern-Volmer plots of hTf quenching in the presence of RA. It is quite apparent that there is an upward curvature at all the temperatures, i.e., there is a positive deviation. By fitting the fluorescence intensity ratio *F*_0_/*F* for different quencher concentration [*C*] (only linear points were considered) in equation (1), Stern-Volmer constant (*K*_sv_) was found at different temperatures, and the obtained *K*_sv_ values are listed in Table SI. *K*_sv_ values were found to decrease with increasing temperature suggesting the operative mode of quenching to be static. However, there was a positive deviation observed in the Stern-Volmer plot, and it can be assumed that both modes of quenching (static and dynamic) are present since the Stern-Volmer plot is linear if one mode of quenching is operative, i.e., either static or dynamic, and positive deviation is observed when quenching is guided by both the modes.

Further, another equation (equation (3)) is used to affirm that hTf-RA is guided by which mode of quenching.

(3) $$\begin{array}{c} K_{q}=\frac{K_{\text{sv}}}{\tau_{0}}. \end{array}$$

The value of the biomolecular quenching rate constant, *K*_*q*_, is implicative of the fact that the process is directed by static or dynamic mode of quenching. Table S1 shows the *K*_*q*_ values obtained using *τ*_o_ (average integral fluorescence lifetime of tryptophan) (2.7 × 10^−9^ s), and these were substantially higher than the maximum dynamic quenching constant (∼10^10^ M^−1^ s^−1^) [37] validating that RA-induced quenching of hTf is due to complex formation. Thus, it can be said that hTf-RA quenching is guided by static mode.

Equation (2) found binding constant (*K*) and other binding parameters for hTf-RA interaction as binding parameters are extremely important in the study of pharmacokinetics and pharmacodynamics of drugs, even the metabolic modification of ligands [38].

Figure 1(b) depicts experimental data fitting as per the modified Stern-Volmer equation; the intercept of this plot gives the value of binding constant (*K*) while the slope gives the number of binding sites (*n*).  Table 1 listed the values of binding constants at different temperatures and suggested that RA binds with an admirable affinity to hTf. Further, it was found that values of *K* were found to decrease with increasing temperature suggesting that a stable complex is formed at lower temperatures. The values of such a high order of binding constant implied that RA would bind to hTf in the circulatory system. Thus, because of all the above observations, it can be concluded that the static mode of quenching is operative for hTf-RA interaction and RA binds to hTf with excellent affinity.

#### 3.1.2. Thermodynamic Parameters of hTf-RA Interaction

Further, to understand the mechanism involved in hTf-RA interaction, thermodynamic parameters were investigated using (Equation (4)) [35], and these parameters can find the forces responsible for the interaction between the protein and ligand. These forces are usually of four types, viz., van der Waals force, electrostatic force, hydrophobic interactions, and hydrogen bonding.

(4) $$\begin{array}{c} \Delta G=-RT\text{ln}K=\Delta H-T\Delta S. \end{array}$$

*K* is the binding constant, Δ*H* is the enthalpy change, Δ*G* is the Gibbs free energy change, Δ*S* is the entropy change, and *R* is the universal gas constant (1.987 cal mol^−1^ K^−1^).

Figure 1(c) gave a linear fit of the obtained data points as per equation (4). The slope of this plot gives the value of Δ*H* and the intercept giving the value of Δ*S*.

The negative value of Δ*G* was obtained for this reaction showing it to be a spontaneous one [39]. For the hTf-RA system, both Δ*H* and Δ*S* are negative, thereby suggesting the hydrogen bonding and van der Waals forces to be the dominant forces in hTf-RA interaction [40].

#### 3.1.3. Isothermal Titration Calorimetry (ITC)

Further, to validate our binding studies through fluorescence spectroscopy, ITC measurements were carried out. A typical isotherm obtained from titration of RA with hTf is depicted in Figure 1(d). The upper part corresponds to raw data obtained as a result of the consecutive injections of RA to hTf. After subtracting the dilution heats of both ligand and protein, binding curves are obtained and shown in the bottom panel. The software that is attached to VP-ITC is Origin 8.0, and this is employed to generate the final figure. The binding curve gives an idea of the heat produced corresponding to each injection as a quantity of the molar ratio of this natural compound (RA) to that of human transferrin. The results presented were obtained from a four-site model of the fitting. Various studies report the difference in values of thermodynamic parameters as obtained from fluorescence spectroscopy and ITC, and this is attributed to the fact that ITC measures a global change in the thermodynamic property whilst fluorescence spectroscopy taking into consideration only the local changes around the fluorophore [41]. Different thermodynamic parameters obtained for hTf-RA interaction as per four model site fitting are *K*_a1_ = 1.15 × 10^5^ ± 8.1 × 10^4^ M^−1^, Δ*H*_1_ = −1345 ± 1.76 × 10^3^ cal/mol, Δ*S*_1_ = 18.6 cal/mol/deg' *K*_*a*2_ = 1.1 × 10^5^ ± 2.4 × 10^4^ M^−1^, Δ*H*_2_ = −3.35 × 10^4^ ± 6.85 × 10^4^ cal/mol, Δ*S*_2_ = −89.4 cal/mol/deg; *K*_*a*3_ = 1.12 × 10^4^ ± 3.7 × 10^4^ M^−1^, Δ*H*_3_ = 7.696 × 10^5^ ± 7.04 × 10^5^ cal/mol, Δ*S*_3_ = 2.6 × 10^3^ cal/mol/deg, *K*_a4_ = −4.91 × 10^6^ ± 5.6 × 10^6^ M^−1^, Δ*H*_4_ = −4.91 × 10^6^ ± 5.6 × 10^6^, ΔS4 = −1.65 × 10^4^ cal/mol/deg.

#### 3.1.4. Molecular Docking Analysis

**Figure 2:**
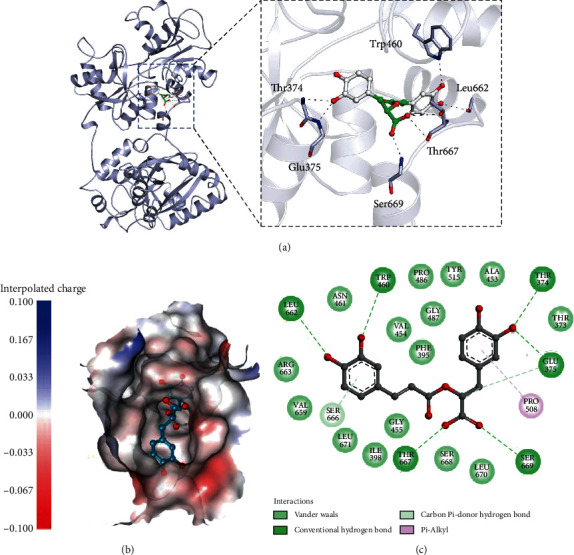
(a) Cartoon representation of hTf in a complex with RA. Three-dimensional view of binding pocket residues of hTf interacting with RA. Polar interactions sharing residues are shown in the sticks (zoomed view). (b) Interpolated charged surface view of hTf binding pocket occupied by RA (the shaded red areas correspond to negatively charged residues and the blue areas to positively charged residues). (c) Two-dimensional diagram of hTf residues interacting with RA.

Molecular docking further provided an insight into the binding affinity of RA to hTf; a significant binding affinity of -8.3 kcal/mol was observed with several close interactions. Molecular docking is a vital method in understanding the foundation of ligand-protein recognition. Several binding pockets were observed in hTf where RA can bind with different conformations; RA is found to bind in the deep cavity most efficiently as depicted in Figure SII. Figure SII shows the RA molecule in the binding pocket of hTf. Many important residues are involved in different types of interaction. hTf is depicted as a cartoon model in light blue while RA is showing in white element, balls, and stick model; binding with many residues that forms hydrogen bonds as depicted by black dashes (Figure 2(a)). A detailed analysis of hTf-RA interaction shows that six residues, viz., Leu662, Trp460, Thr667, Ser669, Glu375, and Thr374 are involved in hydrogen bonding.  Figure 2(b) shows that RA is binding inside the internal pocket of hTf.  Figure 2(b) shows that RA is binding inside the internal pocket of hTf.  Figure 2(c) depicts all the vital interactions for this binding. Thus, molecular docking implied that RA binds in the deep cavity of hTf and hydrogen bonding as the main driving force for this interaction.

### 3.2. Conformational Analysis of RA Binding to hTf

In a bid to investigate the effect of RA on conformation and secondary structure of hTf, FTIR and CD spectroscopy were deployed. Additionally, intrinsic fluorescence and UV spectroscopy gives the clue about changes in the tertiary structure of hTf. Further, MD simulation studies were also performed to find the impact of RA binding on the conformation of hTf and find the stability of the system.

#### 3.2.1. Circular Dichroism (CD) Spectroscopy

**Figure 3:**
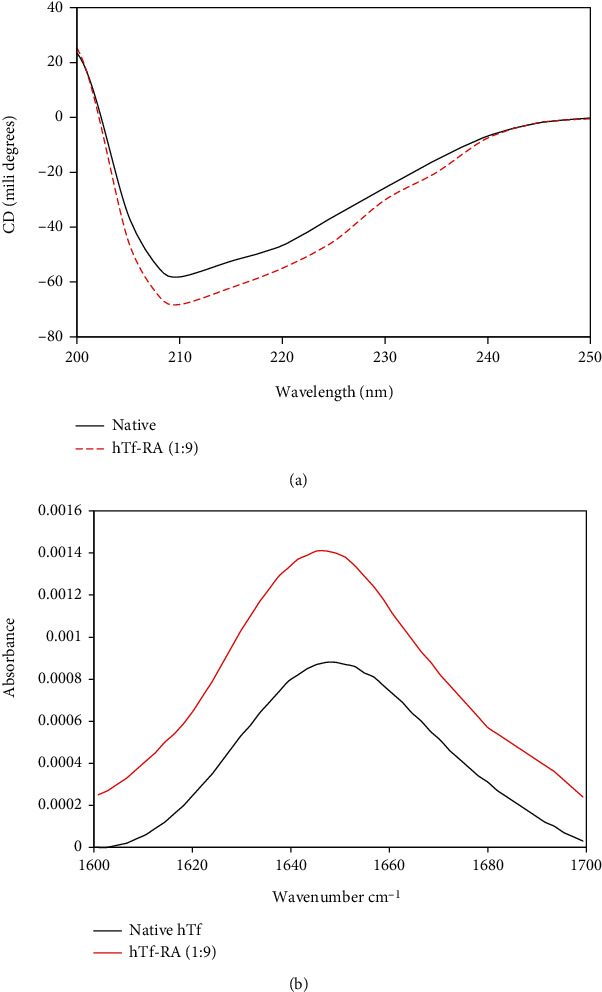
(a) Far-UV CD spectra of the native hTf and hTf-RA (1 : 9) system at room temperature. (b) FTIR spectra of native hTf and hTf-RA with a molar concentration ratio of hTf to RA of 1 : 9.

CD spectroscopy is a technique that is often retorted to characterize conformational alterations occurring in a protein postbinding of a ligand. Changes in the secondary structure of protein correspond to changes in far-UV CD spectra.  Figure 3(a) shows far-UV CD spectra of native hTf and hTf with RA (1 : 9). CD spectra of free hTf showed a peak corresponding to the *α*-helix region, i.e., at around 208 nm and along with a little peak corresponding to *β*-sheet at around 218 nm. With increasing concentration of RA, CD spectra increased in band intensity, i.e., there is a downward shift in the spectrum coupled with no significant shifts in the peak position. This downward movement of CD spectra upon titration with RA indicates that binding of RA induces secondary structural alterations. Several other studies reported that binding of a ligand induces secondary structural alterations in the proteins evident from the same peak position of the free protein and protein-ligand complex but increased/decreased band intensity; thus, our CD observations are in line with those studies [23, 42]. Thus, it can be said that the binding of RA to hTf resulted in secondary structural changes in hTf.

#### 3.2.2. FTIR Spectroscopy

When a ligand binds to the protein, it may cause alterations in the secondary structure of the protein which can be further implicated in functional alterations in protein. Here, we have retorted FTIR spectroscopy to validate our CD spectroscopy results further. Changes in far-UV CD spectra implied secondary structural alterations in hTf in the presence of RA. These changes were further validated by employing FTIR spectroscopy. Amide I is the most intense absorption band in proteins which is mainly decided by stretching vibrations of C=O (70–85%) and C-N groups (10–20%). The frequency for this lies in the range of 1600-1700 cm^−1^ and hence, this is the region of our interest which is investigated for secondary structural alterations.  Figure 3(b) shows the FTIR spectra of native hTf and hTf-RA (1 : 9). It is quite evident that there is a peak shift in the Amide I region from 1649.11 to 1645.253 cm^−1^ which also coupled with a change in intensity. This change in intensity coupled with peak shift demonstrated that RA interacted with hTf inducing secondary structural alterations in native conformation of hTf. Thus, CD spectroscopy coupled with FTIR spectroscopy corroborates our earlier observations and delineated the fact that RA induces secondary structural alterations in native hTf.

#### 3.2.3. Intrinsic Fluorescence Spectroscopy and UV-vis Spectroscopy

To investigate the changes in the local microenvironment in and around aromatic amino acid residues, intrinsic fluorescence spectroscopy is often retorted [43]. Excitation of the protein at 280 nm takes into account all three fluorophores, viz., tryptophan, tyrosine, and phenylalanine while excitation at 295 nm excites only tryptophan residues [44]. Intrinsic fluorescence spectroscopy was carried out by exciting hTf both at 280 nm and 295 nm, and it was found that quenching curves nearly overlap at both these excitation wavelengths at 291 K (Figure SIII) implying that tryptophan was the key player involved in RA-hTf interaction with minimal involvement of other residues.

**Figure 4:**
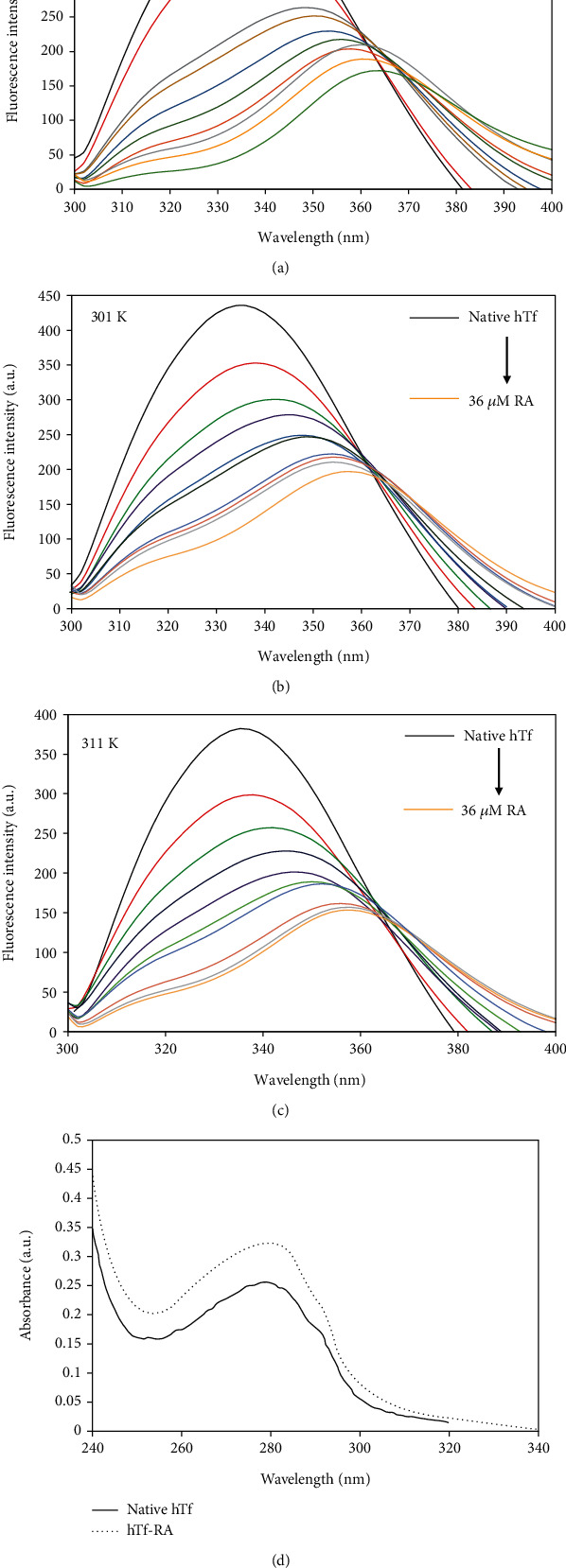
Fluorescence spectra of hTf in the absence and presence of RA (4-36 *μ*M) at (a) 291 K, (b) 301 K, and (c) 311 K. The protein concentration was kept constant at 4 *μ*M while RA was titrated from 4-36 *μ*M. (d) UV spectra of native hTf and hTf-RA (1 : 9).

Figures 4(a)–4(c) depict the fluorescence spectra of native hTf and hTf in the presence of varying RA concentrations (4-36 *μ*M) at three different temperatures. It was apparent that there is a progressive decrease in intrinsic fluorescence at all three temperatures: 291, 301, and 311 K along with a significant redshift (Figures 4(a)–4(c)). There was an appearance of the isoactinic point at around 360 nm implicating the presence of bound and free RA in equilibrium viz., a new complex formation of RA-hTf. These observations suggest that RA interacts with hTf, thereby quenching its intrinsic fluorescence due to RA-hTf complexation. Simultaneously, a redshift was also observed which implies a change in the tertiary structure of hTf in the presence of RA. Thus, it can be concluded that RA induces a change in the secondary and tertiary structures of hTf which can be directly associated with the change in its function.

Figure 4(d) shows the UV spectral profile of native hTf and hTf in the presence of RA. There were significant changes observed in hTf spectra in the presence of RA, thereby suggestive of the fact that RA-hTf interaction was guided by static quenching because dynamic quenching only influenced the excited state of the quenching molecule whilst not affect the absorption spectra of quenching substances [45].

### 3.3. Molecular Dynamic (MD) Simulations

**Figure 5:**
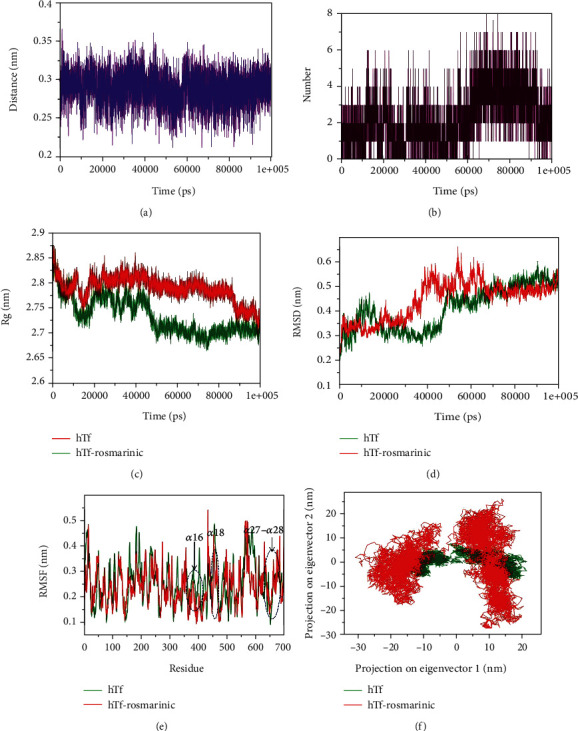
(a) Plot highlighting the changes in the computed distance between the hTf and RA. (b) Hydrogen bond fluctuation curve highlighting the changes in the observed number. (c) The *R*_g_ curves showing the difference in the compactness between the RA bound and unbound hTf. (d) The RMSD plots highlighting the changes between the stabilities in the observed systems. (e) The graphical representation of the changes observed in the fluctuation of the constituent residues between the RA bound and unbound hTf. (f) The 2-D eigenvector projection plot showing the differences between the flexibility of the two studied forms.

To identify the impact of RA binding on the conformation of hTf and validate our in vitro observations, we have performed an extensive MD simulation for both RA bound and unbound forms at a time scale of 100 ns. The RA assumed a closed binding conformation with hTf which was indicative from the computed distance between them which was fluctuated between 0.2-0.35 nm as well as from the number of existed hydrogen bonds with the frequency reaches up to 8 between the protein and inhibitor molecules during MD simulations (Figures 5(a) and 5(b)). The stability of the system was further assessed using the calculated radius of gyration (*R*_g_) and root mean square deviation (RMSD) values, which showed that the system achieved the equilibrium conformation after 50 ns (Figures 5(c) and 5(d)). There is a visible difference existed in the *R*_g_ and RMSD values between the RA bound and unbound forms of the hTf and indicated that the bound form tends to become less stable than the unbound form highlighting the perturbation effect of the RA on the structure of hTf. These observations corroborate CD and FTIR spectroscopy results affirming the fact that binding of RA induces structural alterations in hTf and hence, the hTf-RA complex is less stable as compared to free hTf.

The binding site of the hTf in the docked structure was observed to be at Thr374, Glu375, Trp460, Leu662, Thr667, and Ser669 and the fluctuation in the respective residues between the bound and unbound conformations was also observed (Figure 5(e)). The binding site residues form the corresponding *α*16, *α*18, *α*27, and *α*28 of the secondary structure elements showed relatively lower or comparable motion in the constituent residues in the unbound form of the hTf, indicated the presence of lower or comparable relative energy as compared to the bound form. Furthermore, the flexibility of the conformational states of both the RA bound and unbound forms were assessed using principle component analysis (PCA). It is a statistical technique used for the reduction of the data complexity and is significant in assessing the variation in the atomic motion in biomolecules in the course of MD simulations. A set of eigenvectors and eigenvalues were used to describe the motion of the protein atomic structure. The PCA is significant in the established correlation between the protein functionalities and conformation. The RA bound form occupies a larger conformational space as compared to the unbound conformation of the hTf (Figure 5(f)). This indicated comparatively higher structural motion thus increased dynamics in the essential subspace in the bound form than the unbound form.

**Figure 6:**
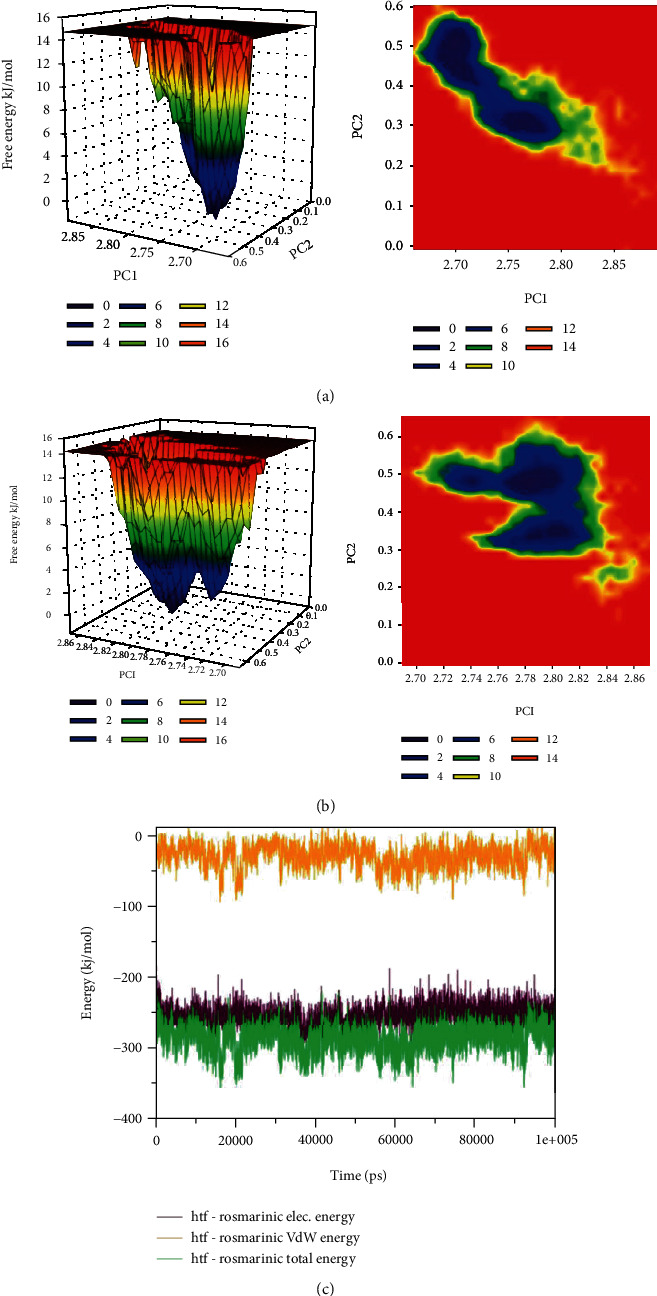
(a) The plots of the free energy landscape and contour map for the unbound form of hTf. (b) The graphical representation of the free energy landscape of the RA bound form. (c) The MMPBSA-based generated curves highlighting the changes in the total, electrostatic, and van der Waals energies calculated between the hTf and RA.

The difference in the folding pattern between the bound and the unbound form of hTf was further studied by analyzing the free energy landscapes of the two conformations. The distinct differences in the free energies were observed between the two conformations (Figure 6(a) and 6(b)). In the RA unbound form of hTf, an energy favored, and relatively stable conformation was observed as compared to the bound form. These observations indicated that the RA binding to the hTf perturb its folding pattern and inhibit the functionality of the protein. Further, the MMPBSA-based algorithms were used for the calculation of the energies present between the hTf and RA in the docked complexes (Figure 6(c)). The total free energy of binding between the protein and the inhibitor was observed around -350 kJ/mol with electrostatic energy that is the major contributor to the binding of the RA with hTf. This observation validated the reliability of the RA binding to the hTf. Thus, molecular dynamic analysis affirmed our in vitro observations concluding that RA binding to hTf results in structural alterations in the protein.

## 4. Conclusion

This study delineates the interaction between hTf and RA employing UV-vis spectroscopy, fluorescence quenching, and CD spectroscopy along with molecular docking and MD simulation studies. Fluorescence quenching studies revealed that the hTf-RA complex is guided by the static mode of quenching. Fluorescence studies were carried out at three temperatures, and the binding constant of hTf-RA was found to be 4.7 × 10^7^ M^−1^ at 291 K implying that RA binds to hTf with a very good affinity. Further, employing the van't Hoff equation, thermodynamic parameters were calculated, and the negative value of ∆*G* showed the reaction to be the spontaneous one. The negative values of ∆*H* and ∆*S* suggested this reaction to be driven by hydrogen bonding and/or van der Waals forces. ITC also advocated the reaction to be spontaneous, i.e., validated our fluorescence spectroscopic results. Further, CD spectroscopy and FTIR spectroscopy were deployed to have an insight into structural alterations in hTF induced by RA, and it was evident from changes in CD and FTIR spectra of the hTf-RA complex as compared to free hTf that binding of RA to hTf induces secondary structural alterations in hTf. Further, molecular docking was also employed to have an insight into the binding mode of RA with hTf. Molecular docking revealed the important residues that are at the heart of this interaction, viz., Leu-662, Trp-460, Thr-667, Ser-669, Glu-375, and Thr-374. Molecular docking also validated our fluorescence studies by confirming the fact that hTf-RA interaction is driven by hydrogen bonding. Further, MD simulation studies were also performed which validated our earlier observations of the hTf-RA complex formation. RA is increasingly being used in the food and cosmetic industries and has several diverse activities, thus making it a significant natural compound to be studied. RA is being used as a food preservative. In Japan, the perilla extracts, rich in rosmarinic acid, are used to garnish and improve the shelf life of fresh seafood. This study elucidated the binding of RA, an important natural compound, with an important plasma protein, hTf, which plays a vital role in neurodegenerative disorders. Many studies have reported the protective role played by polyphenols in neurodegenerative disorders. Also, excessive iron deposition in the central nervous system (CNS) has been linked to neurodegenerative pathologies. It can be said that RA binds strongly to hTf, thereby inducing structural alterations in its native conformation. When a protein loses its native conformation, it can be directly linked to functional alterations. Thus, all the experimental observations support our hypothesis that RA binds with hTf with an excellent affinity and revealed that the hTf-RA complex is guided by the static mode of quenching. Also, conformational changes induced by RA in hTf were confirmed by CD and FTIR spectroscopy which revealed that RA induces secondary structural alterations in hTf. Thus, it can be hypothesized that RA induces structural alterations in hTf such a way that its functionality is compromised leading to lesser iron deposition in CNS and preventing neurodegenerative disorders.

## Figures and Tables

**Table 1:** Thermodynamic parameters obtained for the hTf-RA complex formation.

Temperature (*K*)	*K* (10^7^ M^−1^)	*n*	Δ*G* (kcal mol^−1^)	Δ*S* (cal mol^−1^ K^−1^)	Δ*H* (kcal mol^−1^)	*T*Δ*S* (kcal mol^−1^)
291	4.7	1.5	-9.90993	-93.34	-37.07	-27.1623
301	0.107	1.25	-8.97651			-28.0957
311	0.08	1.20	-8.0431			-29.029

## Data Availability

No data were used to support this study.

## Supplementary Materials

Supplementary Materials Figure SI. (A) Chemical structure of Rosmarinic acid. (B) Surface representation of human transferrin (PDB ID: 3 V83) Figure SII. The RA molecule depicted in the binding pocket of hTf. hTf was shown as a sphere while the RA molecule was shown in balls and stick models. Figure SIII. Quenching curves of RA/hTf at 291 K at two different wavelength Table SI: Thermodynamic parameters of hTf-RA interaction from the Stern-Volmer plot.
