# Supplementary material for: Evaluation of Binding of Rosmarinic Acid with Human Transferrin and Its Impact on the Protein Structure: Targeting Polyphenolic Acid-Induced Protection of Neurodegenerative Disorders
Source: Oxid Med Cell Longev. 2020 Nov 5;2020:1245875. doi: 10.1155/2020/1245875 (PMC7661129; doi:10.1155/2020/1245875)
Supplement: Supplementary Materials — Figure SI. (A) Chemical structure of Rosmarinic acid. (B) Surface representation of human transferrin (PDB ID: 3 V83) Figure SII. The RA molecule depicted in the binding pocket of hTf. hTf was shown as a sphere while the RA molecule was shown in balls and stick models. Figure SIII. Quenching curves of RA/hTf at 291 K at two different wavelength Table SI: Thermodynamic parameters of hTf-RA interaction from the Stern-Volmer plot. [file 1245875.f1.docx]

*Oxidative Medicine and Cellular Longevity*

**Evaluation of binding of Rosmarinic acid with human transferrin and its impact on protein structure: Targeting polyphenolic acid-induced protection of neurodegenerative disorders**

Anas Shamsi^1#^, Saleha Anwar^1#^, Mohd Shahbaaz ^2,3^, Taj Mohammad^1^, ^4^, Mohamed F. Alajmi^4^, Afzal Hussain^4^, Md. Imtaiyaz Hassan^1^, Faizan Ahmad^1^, Asimul Islam^*^

***^1^*** *Centre for Interdisciplinary Research in Basic Sciences, New Delhi, India.*

***^2^*** *South African National Bioinformatics Institute, University of the Western Cape, Private Bag*

*X17, Bellville, Cape Town 7535, South Africa.*

***^3^*** *Laboratory of Computational Modeling of Drugs, South Ural State University, 76 Lenin Prospekt, Chelyabinsk, Russia, 454080.*

*^4^ Department of Pharmacognosy, College of Pharmacy, King Saud University, Riyadh 11451, Saudi Arabia*

*# Both authors contributed equally*

****To whom all correspondence should be addressed,***

**Asimul Islam, Ph.D.**

Centre for Interdisciplinary Research in Basic Sciences

Jamia Millia Islamia, Jamia Nagar

New Delhi 110025, INDIA

Cell: +91-9312812007

E-mail: [aislam@jmi.ac.in](mailto:aislam@jmi.ac.in)


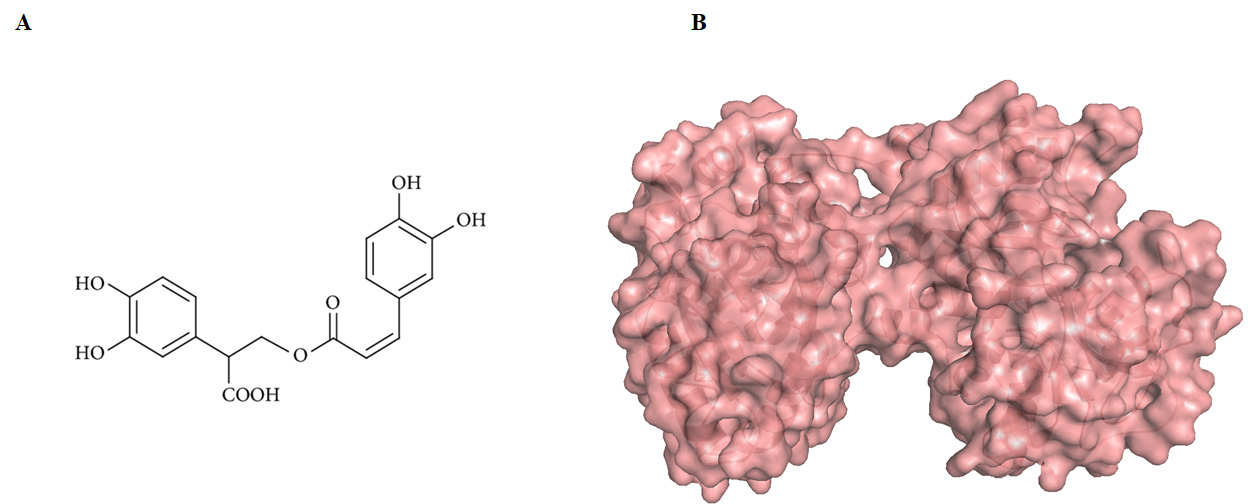


***Figure SI.*** *(A) Chemical structure of Rosmarinic acid. (B) Surface representation of human transferrin (PDB ID: 3V83)*

***TABLE SI: Thermodynamic parameters of hTf-RA interaction from stern volmer plot***

| **Temperature, K** | ***K_sv_* (10^4^ M^-1^)** | ***K*_q_ (10^13^ M^-1^ s^-1^)** | **R^2^** |
| --- | --- | --- | --- |
| 291 | 9.5 | 3.51 | 0.96 |
| 301 | 7.6 | 2.81 | 0.99 |
| 311 | 6.2 | 2.29 | 0.99 |

*
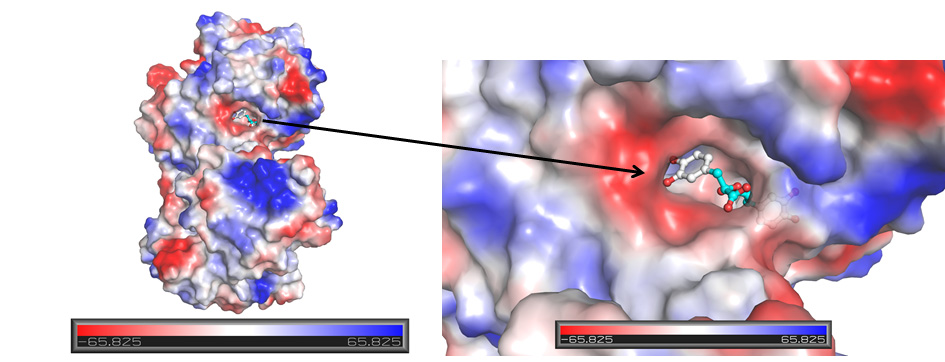
*

***Figure SII.*** *RA molecule depicted in the binding pocket of hTf. hTf was shown as a sphere while RA molecule was shown in balls and stick models.*


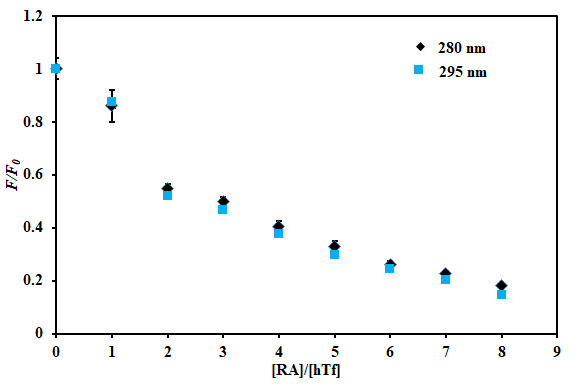


***Figure SIII.*** *Quenching curves of RA/hTf at 291 K at two different wavelength*
